# Willingness to Share Wearable Device Data for Research Among Mechanical Turk Workers: Web-Based Survey Study

**DOI:** 10.2196/19789

**Published:** 2021-10-21

**Authors:** Casey Overby Taylor, Natalie Flaks-Manov, Shankar Ramesh, Eun Kyoung Choe

**Affiliations:** 1 Departments of Medicine and Biomedical Engineering Johns Hopkins University School of Medicine Baltimore, MD United States; 2 Institute for Computational Medicine Johns Hopkins University Baltimore, MD United States; 3 Department of Medicine Division of General Internal Medicine Johns Hopkins University School of Medicine Baltimore, MD United States; 4 College of Information Studies University of Maryland College Park, MD United States

**Keywords:** wearables, personal data, research participation, crowdsourcing

## Abstract

**Background:**

Wearable devices that are used for observational research and clinical trials hold promise for collecting data from study participants in a convenient, scalable way that is more likely to reach a broad and diverse population than traditional research approaches. Amazon Mechanical Turk (MTurk) is a potential resource that researchers can use to recruit individuals into studies that use data from wearable devices.

**Objective:**

This study aimed to explore the characteristics of wearable device users on MTurk that are associated with a willingness to share wearable device data for research. We also aimed to determine whether compensation was a factor that influenced the willingness to share such data.

**Methods:**

This was a secondary analysis of a cross-sectional survey study of MTurk workers who use wearable devices for health monitoring. A 19-question web-based survey was administered from March 1 to April 5, 2018, to participants aged ≥18 years by using the MTurk platform. In order to identify characteristics that were associated with a willingness to share wearable device data, we performed logistic regression and decision tree analyses.

**Results:**

A total of 935 MTurk workers who use wearable devices completed the survey. The majority of respondents indicated a willingness to share their wearable device data (615/935, 65.8%), and the majority of these respondents were willing to share their data if they received compensation (518/615, 84.2%). The findings from our logistic regression analyses indicated that Indian nationality (odds ratio [OR] 2.74, 95% CI 1.48-4.01, *P*=.007), higher annual income (OR 2.46, 95% CI 1.26-3.67, *P*=.02), over 6 months of using a wearable device (OR 1.75, 95% CI 1.21-2.29, *P*=.006), and the use of heartbeat and pulse tracking monitoring devices (OR 1.60, 95% CI 0.14-2.07, *P*=.01) are significant parameters that influence the willingness to share data. The only factor associated with a willingness to share data if compensation is provided was Indian nationality (OR 0.47, 95% CI 0.24-0.9, *P*=.02). The findings from our decision tree analyses indicated that the three leading parameters associated with a willingness to share data were the duration of wearable device use, nationality, and income.

**Conclusions:**

Most wearable device users indicated a willingness to share their data for research use (with or without compensation; 615/935, 65.8%). The probability of having a willingness to share these data was higher among individuals who had used a wearable for more than 6 months, were of Indian nationality, or were of American (United States of America) nationality and had an annual income of more than US $20,000. Individuals of Indian nationality who were willing to share their data expected compensation significantly less often than individuals of American nationality (*P*=.02).

## Introduction

A wearable device is a small hardware technology that people wear on various parts of their bodies. These devices’ functions may include tracking and monitoring health, fitness, food, and aging-related metrics [1]. Using wearables has become very popular in part because data collection and presentation occur in real time [2] and due to their potential for having a positive effect on health and fitness [3]. In addition, regular feedback provided by wearables shows promise for positively influencing physical activity and weight loss outcomes [4]. From a research perspective, wearables hold promise for collecting data from study participants in a convenient, scalable way that is more likely to reach a broad and diverse population than traditional research approaches [5,6].

Previous research on wearable device data sharing for research has yielded mixed results regarding the willingness to share such data [7-9]. Amazon Mechanical Turk (MTurk) is a potential resource that researchers can use to recruit individuals into studies that use data from wearables. MTurk is a crowdsourcing platform that allows researchers to post human intelligence tasks (HITs) that “workers” can choose to complete for compensation [10]. Previous studies have assessed the opinions of individuals from targeted populations such as older adults [7], health app users [8], and patients [9]. Although there have been studies that have recruited wearable users from MTurk [11,12], to our knowledge, these participants’ characteristics have not been examined. The objective of this study was to explore the characteristics of wearable device users on MTurk that are associated with a willingness to share data for research use. We also aimed to determine whether compensation was a factor that influenced the willingness to share such data. This study will help researchers who are considering MTurk as a research avenue to recruit wearable device users from MTurk and to understand the potential benefits and limitations.

## Methods

### Study Design and Population

This was a secondary analysis of a cross-sectional survey study of MTurk workers who use wearable devices for health monitoring. These workers participated in the survey from March 1 to April 5, 2018. Only adults over the age of 18 years were eligible for this study. Details on the recruitment strategy and survey instrument were reported in the primary publication [13] and are summarized in the *Recruitment Strategy and Survey Instrument* section.

### Recruitment Strategy and Survey Instrument

We developed a 19-question web-based survey to explore the characteristics that are associated with a willingness to share wearable device data for research use. This survey was prepared by using Qualtrics (Qualtrics International Inc). After publishing a short description of the survey to the MTurk interface (ie, a HIT), MTurk workers who met the eligibility requirement were able to review the HIT, and if they chose to undertake the survey task, they were referred to the external Qualtrics survey website (Multimedia Appendix 1).

On the Qualtrics survey website, the web-based recruitment strategy involved four parts—an introduction to this study, screening questions, survey questions, and postsurvey steps. The introduction provided an overview of this study, which included details about the time limit, compensation, risks, benefits, and the contact details of the investigators. The screening portion was included to make sure that the respondents were qualified to take the survey and that they understood important concepts. Comprehension was assessed by using multiple-choice questions on the meanings of *health monitoring technology* and *health monitoring data* after providing descriptions. In order to assess whether a respondent was qualified to take the survey, they were asked to indicate if they use a wearable device to monitor their health (Multimedia Appendix 1). Only participants who answered the comprehension questions correctly and indicated that they use a wearable to monitor their health could move on to complete the survey. The survey included questions about demographics, experience with MTurk, motivations for participating in MTurk HITs, experience with using wearables, and interest in submitting wearable device data for research purposes. For the postsurvey steps, upon the successful completion of the survey, respondents were given a short message of appreciation for their participation. They were also provided with a random, automatically generated validation code. In order to receive payment for completing the survey, the validation code had to be entered on MTurk. Each participant was paid US $0.40 for their responses.

Pilot tests for this process were conducted to determine the time required for completing the survey. Additionally, to make sure that the efficiency of the data did not decrease, we ensured that respondents from all of the previous batches were unable to respond to the latest batch of HITs.

This study was judged as one that imposed only minimal risks on participants and was determined to be exempt from institutional review by both the Johns Hopkins University (protocol code: IRB00158371) and University of Maryland (protocol code: IRB 1165377) institutional review boards.

### Outcomes and Associated Variables

The primary outcome was a willingness to share wearable device data for research purposes. The secondary outome was a willingness to share wearable device data if compensation is provided. The focus of the analysis was to understand the characteristics associated with a willingness to share wearable device data and those associated with a willingness to share such data if compensation is provided. The surveyed demographic characteristics included age (18-24 years, 25-34 years, 35-44 years, and ≥45 years), sex (male and female), nationality (American [United States of America], Indian, and other), annual income in US dollars (<US $20,000; US $20,000-US $39,999; US $40,000-US $69,999; and ≥US $70,000), and education (high school diploma or other, some college, a bachelor’s degree, and a graduate degree or work). In addition, the surveyed wearable device characteristics included the purpose of use (heartbeat and pulse tracking; sleep tracking; step tracking; and diet-related tracking, that is, the tracking of calories, body fat, or nutrients) and the duration of use (<6 months and ≥6 months). We also tested for a possible association between the willingness to share wearable device data and the average time spent on doing HITs per week (0 to <2 hours, 2 to <4 hours, 4 to <8 hours, 8 to <20 hours, and ≥20 hours).

### Statistical Analysis

Bivariate analyses via chi-square tests were used for categorical variables to estimate differences in characteristics between workers with and without a willingness to share wearable device data for research use. Similar analyses were performed for workers who were willing to share such data to estimate differences in characteristics between those who were expecting compensation and those who were not. With regard to multivariable analyses, we performed a logistic regression to evaluate the relationship between a willingness to share wearable device data and the individual characteristics of MTurk workers. A similar model was used to analyze the willingness to share such data if compensation is provided. In addition, we performed decision tree analyses to identify subgroups that have a higher probability to share wearable data for research. All analyses were performed by using R version 3.6.2 (R Foundation for Statistical Computing).

## Results

A total of 935 MTurk workers who use wearable devices completed the survey. Almost 90% (827/935, 88.4%) of participants were aged 18 to 44 years, 58.9% (551/935) were male, 64.7% (605/935) were of American nationality, and about 60% (578/935, 61.8%) had been using wearables for more than 6 months (Table 1). Two-thirds of all respondents (615/935, 65.8%) indicated a willingness to share their wearable device data. Nationality, annual income, education, the average time spent on completing HITs per week, the duration of using wearable devices, and the use of wearables that track heartbeat and pulse had a statistically significant association with a willingness to share data without expecting compensation in the univariate analysis (Table 1).

**Table 1:** Univariate analysis of the association between the willingness to share wearable device data for research and Amazon Mechanical Turk workers’ characteristics and between the willingness to share data if compensation is provided and workers’ characteristics.

Characteristics		Total (N=935), n (%)	Willingness to share wearable device data			Willingness to share wearable device data if compensation is provided^a^		
			No (n=320), n (%)	Yes (n=615), n (%)	*P* value	No (n=97), n (%)	Yes (n=518), n (%)	*P* value
**Sex**								
	Male	551 (58.9)	188 (58.8)	363 (59)	N/A^b^	62 (63.9)	301 (58.1)	N/A
	Female	375 (40.1)	129 (40.3)	246 (40)	.97	33 (34)	213 (41.1)	.27
	Other or prefer not to say	9 (1)	3 (0.9)	6 (1)	N/A	2 (2.1)	4 (0.8)	N/A
**Age (years)**								
	18-24	130 (13.9)	52 (16.3)	78 (12.7)	N/A	14 (14.4)	64 (12.4)	N/A
	25-34	490 (52.4)	159 (49.7)	331 (53.8)	.59	49 (50.5)	282 (54.4)	.83
	35-44	207 (22.1)	70 (21.9)	137 (22.3)	N/A	24 (24.7)	113 (21.8)	N/A
	≥45	100 (10.7)	37 (11.6)	63 (10.2)	N/A	10 (10.3)	53 (10.2)	N/A
	Prefer not to say	8 (0.9)	2 (0.6)	6 (1)	N/A	0 (0)	6 (1.2)	N/A
**Nationality**								
	American (United States of America)	605 (64.7)	222 (69.4)	383 (62.3)	N/A	45 (46.4)	338 (65.3)	N/A
	Indian	210 (22.5)	53 (16.6)	157 (25.5)	.008	43 (44.3)	114 (22)	<.001
	Other	120 (12.8)	45 (14.1)	75 (12.2)	N/A	9 (9.3)	66 (12.7)	N/A
**Annual income (US $)**								
	<20,000	226 (24.2)	92 (28.7)	134 (21.8)	N/A	35 (36.1)	99 (19.1)	N/A
	20,000-39,999	236 (25.2)	82 (25.6)	154 (25)	.03	20 (20.6)	134 (25.9)	.01
	40,000-69,999	276 (29.5)	94 (29.4)	182 (29.6)	N/A	26 (26.8)	156 (30.1)	N/A
	≥70,000	194 (20.7)	52 (16.2)	142 (23.1)	N/A	16 (16.5)	126 (24.3)	N/A
	Prefer not to say	3 (0.3)	0 (0)	3 (0.5)	N/A	0 (0)	3 (0.6)	N/A
**Education**								
	Graduate degree or work	192 (20.5)	62 (19.4)	130 (21.1)	N/A	19 (19.6)	111 (21.4)	N/A
	Bachelor’s degree	472 (50.5)	147 (45.9)	325 (52.8)	.04	53 (54.6)	272 (52.5)	.90
	Some college	210 (22.5)	83 (25.9)	127 (20.7)	N/A	21 (21.6)	106 (20.5)	N/A
	High school diploma or other	61 (6.5)	28 (8.8)	33 (5.4)	N/A	4 (4.1)	29 (5.6)	N/A
**Average time spent on doing human intelligence tasks per week (hours)**								
	0 to <2	120 (12.8)	46 (14.4)	74 (12)	N/A	12 (12.4)	62 (12)	N/A
	2 to <4	183 (19.6)	77 (24.1)	106 (17.2)	.04	15 (15.5)	91 (17.6)	.45
	4 to <8	225 (24.1)	63 (19.7)	162 (26.3)	N/A	31 (32)	131 (25.3)	N/A
	8 to <20	228 (24.4)	75 (23.4)	153 (24.9)	N/A	18 (18.6)	135 (26.1)	N/A
	≥20	179 (19.1)	59 (18.4)	120 (19.5)	N/A	21 (21.6)	99 (19.1)	N/A
**Duration of using wearable devices (months)**								
	≤6	357 (38.2)	150 (46.9)	207 (33.7)	N/A	48 (49.5)	159 (30.7)	N/A
	>6	578 (61.8)	170 (53.1)	408 (66.3)	<.001	49 (50.5)	359 (69.3)	.001
**Purpose of wearable device^c^**								
	Heartbeat and pulse tracking	446 (47.7)	131 (40.9)	315 (51.2)	.004	49 (50.5)	266 (51.4)	.97
	Sleep tracking	323 (34.5)	103 (32.2)	220 (35.8)	.31	32 (33)	188 (36.3)	.61
	Step tracking	648 (69.3)	218 (68.1)	430 (69.9)	.62	54 (55.7)	376 (72.6)	.001
	Diet (calories, body fat, and nutrition)	552 (59)	180 (56.2)	372 (60.5)	.24	61 (62.9)	311 (60)	.68

^a^Data are from those who are willing to share data.

^b^N/A: not applicable.

^c^More than 1 option could be selected by the same person.

Among those who were willing to share their wearable device data, 84.2% (518/615) indicated that they were willing to do so if compensation was provided. Nationality, annual income, the duration of using wearable devices, and the use of wearables that track steps had a significant univariate association with a willingness to share data if compensation is provided. A more granular breakdown of the duration of using wearable devices among our study population is shown in Multimedia Appendix 2.

The findings from our logistic regression analyses indicated adjusted associations between the willingness of MTurk workers to share wearable device data and their characteristics. In particular, Indian nationality (odds ratio [OR] 3.09, 95% CI 1.92-5.02); annual incomes of US $20,000-US $39,999, US $40,000-US $69,999, and ≥US $70,000 (OR 1.61, 95% CI 1.04-2.52; OR 1.73, 95% CI 1.12-2.7; OR 2.32, 95% CI 1.4-3.87, respectively); over 6 months of using a wearable (OR 1.74, 95% CI 1.26-2.4); and the use of heartbeat and pulse tracking wearables (OR 1.58, 95% CI 1.17-2.14) were associated with a higher willingness to share data for research use (Table 2).

**Table 2:** Multivariable analysis (logistic regression) of the association between the willingness of Amazon Mechanical Turk workers to share wearable device data for research and their characteristics and between their willingness to receive compensation for donating wearable device data and their characteristics.

Characteristics				Willingness to share wearable device data				Willingness to share wearable device data if compensation is provided		
				Odds ratio (95% CI)		*P* value		Odds ratio (95% CI)		*P* value
**MTurk workers’ characteristics**										
	**Sex**									
		Male	1.00 (reference)		Reference		1.00 (reference)		Reference	
		Female	1.10 (0.78-1.43)		.53		1.17 (0.72-1.92)		.53	
	**Age (years)**									
		18-24	1.00 (reference)		Reference		1.00 (reference)		Reference	
		25-34	0.99 (0.56-1.42)		.96		1.12 (0.54-2.24)		.75	
		35-44	0.97 (0.50-1.45)		.91		0.74 (0.33-1.6)		.45	
		≥45	0.88 (0.37-1.39)		.64		0.78 (0.3-2.1)		.62	
	**Nationality**									
		American	1.00 (reference)		Reference		1.00 (reference)		Reference	
		Indian	2.74 (1.48-4.01)		.007		0.47 (0.24-0.9)		.02	
		Other	1.13 (0.64-1.62)		.61		1.16 (0.54-2.74)		.71	
	**Annual income (US $)**									
		<20,000	1.00 (reference)		Reference		1.00 (reference)		Reference	
		20,000-39,999	1.61 (0.93-2.29)		.08		1.72 (0.88-3.43)		.11	
		40,000-69,999	1.76 (1.02-2.51)		.045		1.31 (0.68-2.55)		.42	
		≥70,000	2.46 (1.26-3.67)		.02		1.52 (0.71-3.32)		.29	
	**Education**									
		Graduate degree or work	1.00 (reference)		Reference		1.00 (reference)		Reference	
		Bachelor’s degree	1.31 (0.81-1.81)		.23		0.97 (0.52-1.78)		.93	
		Some college	1.10 (0.60-1.60)		.69		0.64 (0.29-1.39)		.27	
		High School diploma or other	0.93 (0.32-1.54)		.82		1.03 (0.31-4.13)		.96	
	**Average time spent on doing human intelligence tasks per week (hours)**									
		0 to <2	1.00 (reference)		Reference		1.00 (reference)		Reference	
		2 to <4	0.73 (0.37-1.10)		.15		1.22 (0.51-2.88)		.65	
		4 to <8	1.41 (0.71-2.11)		.25		0.89 (0.4-1.88)		.76	
		8 to <20	1.16 (0.59-1.73)		.58		1.61 (0.69-3.66)		.26	
		≥20	1.16 (0.56-1.76)		.60		1.25 (0.54-2.83)		.59	
**Health monitoring technologies**										
	**Duration of using wearable devices (months)**									
		≤6	1.00 (reference)		Reference		1.00 (reference)		Reference	
		>6	1.75 (1.21-2.29)		.006		1.60 (0.99-2.6)		.06	
	**Purpose of wearable device^a^**									
		Heartbeat and pulse tracking	1.60 (1.14-2.07)		.01		1.07 (0.67-1.71)		.77	
		Sleep tracking	1.11 (0.77-1.44)		.54		1.00 (0.62-1.65)		.99	
		Step tracking	1.17 (0.77-1.57)		.41		1.45 (0.85-2.44)		.17	
		Diet (calories, body fat, and nutrition)	1.09 (0.77-1.41)		.60		0.96 (0.59-1.57)		.88	

^a^More than 1 option could be selected by the same person.

**Figure 1:**
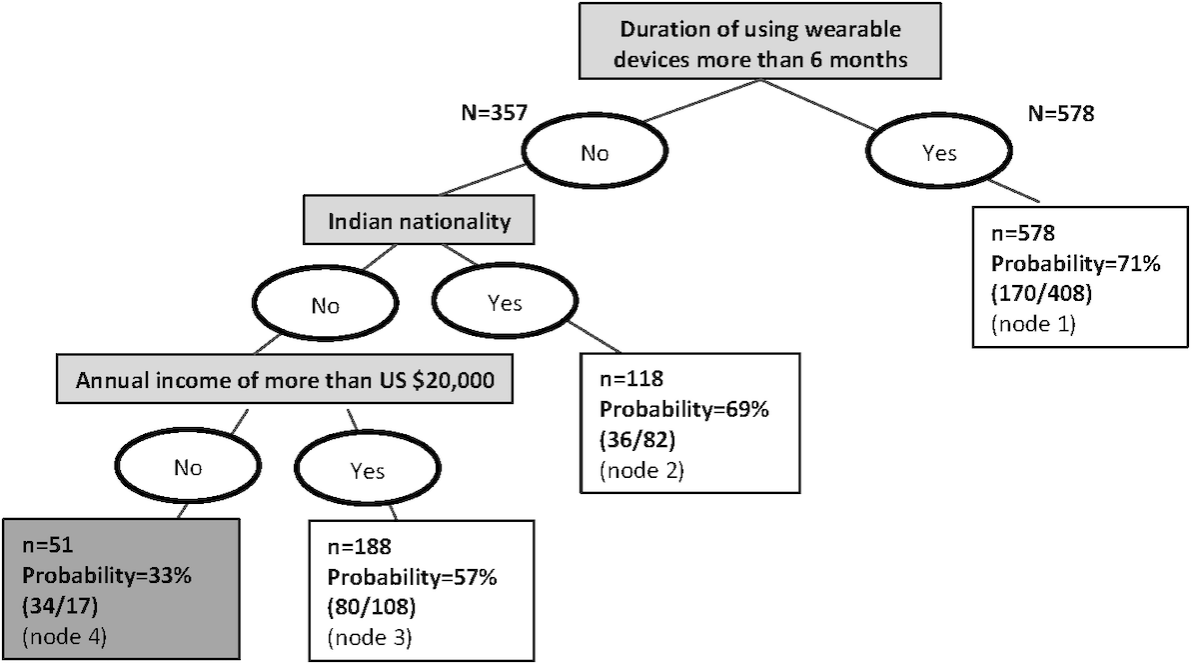
Decision tree analysis for the classification of Amazon Mechanical Turk workers who are willing or not willing to share wearable device data for research purposes (N=935; Accuracy=0.684).

The decision tree analysis identified three characteristics that were significantly associated with the willingness to share wearable data for research and subdivided all participants into 4 segments (nodes). The first characteristic in the tree (highest importance) was the use of health monitoring wearables for more than 6 months. If the response was positive (ie, “yes”; node 1; n=578), these participants had a 71% probability of having a willingness to share wearable data. For participants who indicated that they have been using wearables for 6 months or less, the second question was “what is your nationality?” If they were of Indian nationality (node 2; n=118), then the probability of having a willingness to share wearable data was 69%. If the participant was not Indian, the next question was “what is your annual income?” Individuals with an annual income of more than US $20,000 (node 3; n=188) had a 57% probability of having a willingness to share wearable data. The last segment of participants, who indicated a wearable device use duration of less than 6 months, were not Indian, and had an annual income of less than US $20,000 (node 4; n=51), had a low probability of being willing to share wearable data (33%). The accuracy of the decision tree was 0.684 (Figure 1).

## Discussion

### Principal Findings

In this study, we explored the characteristics of wearable device users on MTurk that are associated with a willingness to share wearable device data for research use. Our findings show that about two-thirds of individuals (615/935, 65.8%) who used wearables indicated a willingness to share their data for research use. The probability of having a willingness to share wearable device data was higher among individuals who had been using wearables for more than 6 months, individuals with higher incomes, individuals of Indian nationality, and individuals who use wearables for heartbeat and pulse tracking. The majority of those who were willing to share wearable data (518/615, 84.2%) preferred to receive compensation for sharing such data. Further, MTurk workers from India expected compensation significantly less often than American workers (*P*=.02). No other factors were associated with the willingness to share wearable device data if compensation is provided.

Our principal finding is that most MTurk workers (615/935, 65.8%) were willing to share their wearable data for research; however, the results of similar studies based on different populations are mixed. One study of older adults [7] and another study of health and fitness app users [8] found that a majority of participants were willing to share their digital device data. The study with older adults also found an association between income and a willingness to share such data [7]. In another study of patients who were seeking care in an academic emergency department, a minority (40%) of patients indicated a willingness to share wearable device data with researchers [9]. More patients were willing to share other types of data (eg, music streaming data), and a majority were willing to share all surveyed digital data types after death, including wearable device data.

Our finding that a willingness to share wearable device data may differ due to experience with using such devices is similar to the findings of others. For example, a survey found that those with a self-rated low or medium level of expertise with wearables were less willing to share such data [14]. Another survey of health and fitness app users found that “quantified-selfers” were significantly more willing to share their personal data on a public scientific database compared to “non–quantified-selfers” [8]. The relationship between experience and the willingness to share data may be influenced by factors such as a knowledge of data protection laws and people’s comfort with the privacy implications of sharing data. Furthermore, individuals who have more experience with engaging in self-tracking or data sharing activities may be different from individuals with less experience (eg, being more accustomed to sharing data due to previously participating in self-tracking studies or finding value in sharing and discussing their data with others as part of the quantified self movement).

This study is among the few investigating the willingness to share wearable device data if financial compensation is provided. In a review of motivating factors that influence participation in genomic studies, the authors found compensation to be the least important factor [15]. Genomic studies however largely do not involve wearable data donation. In another study that had a sample of individuals that was more similar to ours (ie, a population in which self-tracking was common), the authors found that a majority “probably would” or “definitely would” be willing to share their data and that a majority would be “more” or “much more” willing to share data if they were compensated [16]. Different from that study, we also investigated characteristics that are associated with the willingness to share if compensation is provided and found nationality to be the only statistically significant factor (*P*=.02).

We also found that people who collect heartbeat and pulse tracking data are more willing to share wearable device data. When considering the two data representation levels (ie, sensor data and derived information [14]) that were present among the data types covered in our survey, heartbeat and pulse tracking data were the only data type at the sensor data level. The other data types were derived information from accelerometors (ie, those for sleep tracking and step tracking) or other sensors (ie, those for tracking calories, body fat, nutrients). This finding might be suggestive of a lower concern with data privacy, given that derived information only makes use of a subset of the available raw data. However, given that we did not make the distinction between raw and derived data in our survey, it is very possible that survey respondents were unaware of the kinds of derived information that can be collected from heartbeat and pulse tracking devices (eg, health status and life expectations). According to the work of Schneegass et al [14], wearable device users’ understanding of the relationship between sensor data and the derived information from sensor data is still limited. Thus, when considering mechanisms for increasing people’s willingness to share wearable device data, requesting access to derived information may be the most appropriate approach for maximizing the transparency on data that would be studied in the research.

The demographic characteristics of the MTurk workers who enrolled in this study were in close accord with those in another study that was conducted at around the same time [17]. Most of the workers were from the United States (605/935, 64.7% in this study vs the 75% in the work of Difallah et al [17]), the second largest group was from India (210/935, 22.5% in our survey vs the 16% in the work of Difallah et al [17]), and the remaining respondents were from other countries. Males constituted 58.9% (551/935) of the respondents in our study. This is similar to what we have observed among MTurk workers for most countries except the United States [17,18]. The population of MTurk workers who use wearable devices is slightly younger than the general population of MTurk workers. Almost 90% (827/935, 88.4%) of our population were aged 18 to 45 years, whereas 70% of general MTurk workers are aged 18 to 50 years [17]. The age distribution in our study however is similar to that of wearable device users in the United States [19]. The annual income of our population was lower than that of the general US population [20] but was similar to the income of MTurk workers [17]. The median income of the study population was below US $40,000, whereas the median income of MTurk workers is about US $47,000 and the median income of the US general population is about US $60,000.

### Limitations

First, the generalizability of MTurk survey responses is a common concern for researchers. Our study found some similarities to and some differences from the general population of wearable device users. Our sample may also have been more driven by compensation than a general study population due to our use of MTurk. Future work might compare the MTurk sample with other types of samples, such as web panels [21] and social media forum users who are not offered compensation. Second, data quality concerns are also common with crowdsourcing studies such as ours. In order to improve the quality of our data, we built a comprehension screening test into our survey. Although it was not explored in this study, high data quality may also be better ensured through the use of sampling strategies that take the experience and reputation of workers into consideration [22]. Third, opinions about a willingness to share wearable device data may differ from actual decisions. This has been observed in a study of the willingness to participate in biobank research [23]. Similar factors that influence participation in biobank research that are less influential in a hypothetical context, such as trust, may play a part in whether data are actually shared for research. Others have found that a willingness to share digital data, for example, may be a function of people’s trust in scientific teams [24]. Additionally, informed consent for data donation in situations where individuals may no longer have control over their data after the donation has been completed was not explicitly described to the participants in this study, and this may have influenced their actual willingness. Fourth, the complexity and the multiplicity of influences on wearable data donation could not be fully captured by this study. For example, the demographic characteristics of individuals that were related to a higher probability of having a willingness to share wearable data were high income and Indian nationality. There are likely cultural contexts that are relevant to these factors, such as social norms and the data protection policies of different countries, which influence attitudes toward data donation. Future works that recruit participants by using the MTurk platform should explore other factors that may influence the decision to share data, such as trust; control over what and how data are used; informed consent considerations (eg, broad consent, dynamic consent, etc); and interactions between individual factors and possible broader factors, such as social norms and data protection policies. Furthermore, a better understanding of the extent to which the openness to data sharing translates into actual behaviors exhibited by the broad population of wearable device users will help to guide researchers seeking to recruit these individuals. A person’s comfort with sharing data prior to a data request, for example, may influence the extent to which the hypothetical willingness to share data translates into an actual data donation.

### Implications

#### Crowdsourcing Wearable Device Data Donation

This study summarizes the characteristics of potential wearable users who were recruited from MTurk. Missing descriptions of crowd workers’ characteristics in study reports are a frequent issue in health research studies [25]. Although there are other studies that have recruited wearable users from MTurk [11,12], to our knowledge these workers’ characteristics have not been examined. Once participants give approval for accessing their wearable device data, mechanisms for granting access, such as on-device permissions (eg, as with Apple HealthKit) and the OAuth 2.0 protocol [26], can be used to allow researchers to remotely access wearable device users’ data. Therefore, the crowdsourcing aspect of MTurk can allow researchers to quickly reach a large number and a diversity of study participants [10]. As MTurk is considered further as a source for recruiting study participants who are willing to share wearable device data, the secondary use of such data for research will require the careful consideration of current data protection laws, such as the European Union General Data Protection Regulation and US Health Insurance Portability and Accountability Act.

#### Enabling Wearable Data Donation

In order to enable wearable device data donation by MTurk workers, technical approaches are needed to obtain permissions for the secondary use of data and for transferring data to research platforms that have the appropriate levels of privacy and security. There are platforms, such as mCerebrum DataKit [27] and Open Humans [28], that have been designed to collect mobile sensor data from multiple sources, including wearable devices. However, there are few devices that support the transfer of data [29]. A recent review of iOS personal health apps showed that only 33% of apps support application programming interfaces—a data access method that allows for the most fine-grained data access [26]. Given the high willingness of MTurk workers to share wearable device data that was identified in our study, future work might enable wearable data access for devices that are commonly used by MTurk workers and establish opt-in data collection services that are compatible with existing platforms.

### Conclusions

In this study, we examined MTurk workers’ willingness to share wearable device data for research use and the characteristics associated with a willingness to share such data. We found that about two-thirds of wearable device users on MTurk (615/935, 65.8%) indicated a willingness to share their data for research use. Among those who were willing to share such data, most (518/615, 84.2%) indicated that they were willing to share their data if compensation is provided. The probability of having a willingness to share wearable device data was higher among individuals who had used a wearable for more than 6 months, were of Indian nationality, or were of American nationality and had an annual income of more than US $20,000. Overall, our findings are encouraging and should be considered by crowdsourcing research studies that involve wearable device data sharing. Existing platforms for opt-in data collection should be used to achieve this goal.

## Appendix

Multimedia Appendix 1 Human intelligence task description and comprehension check question.

Multimedia Appendix 2 The duration of wearable device use among Amazon Mechanical Turk survey participants.

## Abbreviations

HIT: human intelligence task
MTurk: Amazon Mechanical Turk
OR: odds ratio
