# Supplementary material for: Willingness to Share Wearable Device Data for Research Among Mechanical Turk Workers: Web-Based Survey Study
Source: J Med Internet Res. 2021 Oct 21;23(10):e19789. doi: 10.2196/19789 (PMC8569545; doi:10.2196/19789)
Supplement: Multimedia Appendix 1 [file jmir_v23i10e19789_app1.docx]

**Appendix 1.**

**Appendix 1a.** Human Intelligence Task (HIT) description

Have you ever used a Health monitoring technology?

If yes, you are eligible for this survey.

This survey aims at understanding the demographics of Amazon Mechanical Turk participants who use Health monitoring technologies. It should only take about 3 to 5 minutes.

After completion of this survey, you will receive a Validation code that must be entered back on the Mechanical Turk webpage to claim your compensation. You will be awarded $0.40 for your time. Be assured that all answers provided will be strictly confidential. Your participation in this research is completely voluntary.

Go to the *(MTurk survey link)* to learn more about the study and participate.

**Appendix 1b.** Comprehension check question

**Health monitoring technology:** A health monitoring technology is a wearable (e.g., wristband, clip-on), stand-alone device, or mobile app used for monitoring and tracking health and fitness-related metrics. Examples of the metrics include any of the following:

1. steps, distance walked, or run

2. food, calorie consumption, nutritional consumption

3. physiological functions like heartbeat, pulse rate, blood pressure

4. sleep duration, sleep quality

**Health monitoring data:** Health monitoring data are data produced by a health monitoring technology.

According to the above descriptions which, if any, of the following would be considered health monitoring technologies? (select all that apply)

- Glucose meter
- Activity Tracker (e.g., Fitbit)
- Thermometer
- Weighing Scale
