# Supplementary material for: Willingness to Share Wearable Device Data for Research Among Mechanical Turk Workers: Web-Based Survey Study
Source: J Med Internet Res. 2021 Oct 21;23(10):e19789. doi: 10.2196/19789 (PMC8569545; doi:10.2196/19789)
Supplement: Multimedia Appendix 2 [file jmir_v23i10e19789_app2.docx]

**Appendix 2:** Duration of wearable device use among MTurk survey participants

| **Time of use** | **Total** |
| --- | --- |
|  | **935** |
| 0-3 months | 177 (18.9%) |
| 4-6 months | 180 (19.3%) |
| 7-9 months | 108 (11.6%) |
| 10-12 months | 99 (10.6%) |
| 12+ months | 371 (39.7%) |
